#  Cell Kinetics of Regenerating Liver After 70% Hepatectomy in Rats - 2-Color Flow Cytometric Analysis

**DOI:** 10.1155/1992/51372

**Published:** 1992

**Authors:** Jun Tamura, Junji Tanaka, Ken-Ichi Fujita, Masanori Yoshida, Takayuki Kasamatsu, Shigeki Arii, Takayoshi Tobe

**Affiliations:** The First Department of Surgery Faculty of Medicine Kyoto University 54 Shogoin kawara-cho, Sakyo-ku Kyoto Japan

## Abstract

Two-color flow cytometric (FCM) analysis using anti-bromodeoxyuridine (BrdU) monoclonal antibody (MoAb) was used to investigate the cell kinetics of regenerating liver after 70% partial hepatectomy in rats. Three peaks were seen in DNA histograms of rat hepatocyte nuclei, corresponding to diploid(2c), tetraploid(4c), and octaploid(8c). These proportions changed in the course of regeneration which were clearly demonstrated by DNA histograms using flow cytometry. The proportion of diploid, tetraploid, and octaploid nuclei in control liver were 49.3 ± 1.6%, 45.0 ± 7.4%, and 1.7 ± 0.7%, respectively. A significant change occurred at 24 hours after hepatectomy, as FCM revealed 25.9 ± 1.1% diploid, 54.5 ± 1.2% tetraploid, and 9.0 ± 0.9% octaploid. This shift to polyploid nuclei persisted until 72 hours, and then gradually returned to the pattern of control liver. The S-phase nuclei which incorporated BrdU increased rapidly at 24 hours to a peak of 11.3 ± 0.9%, and gradually decreased to 5.8 ± 0.8%, 5.3 ± 0.8%, 2.4 ± 0.6%, 2.9 ± 1.1%, and 1.2 ± 0.6%, at 48, 72, 96, 120, and 168 hours, respectively. This 2-color FCM analysis made a detailed analysis of the cell kinetics in regenerating hepatocytes possible, and may be applied in investigations of various aspects of liver regeneration.


*HPB Surgery*, 1992, Vol. 5, pp. 103–115
Reprints available directly from the publisher
Photocopying permitted by license only

© Harwood Academic Publishers GmbH
Printed in the United Kingdom

### CELL KINETICS OF REGENERATING LIVER AFTER 70% HEPATECTOMY IN RATS - 2-COLOR FLOW CYTOMETRIC ANALYSIS

JUN TAMURA, JUNJI TANAKA, KEN-ICHI FUJITA, MASANORI YOSHIDA, TAKAYUKI KASAMATSU, SHIGEKI ARII and TAKAYOSHI TOBE

*The First Department of Surgery, Faculty of Medicine, Kyoto University, 54 Shogoin kawara-cho, Sakyo-ku, Kyoto, Japan*

*(Received 25 February 1991)*

Two-color flow cytometric (FCM) analysis using anti-bromodeoxyuridine (BrdU) monoclonal antibody (MoAb) was used to investigate the cell kinetics of regenerating liver after 70% partial hepatectomy in rats. Three peaks were seen in DNA histograms of rat hepatocyte nuclei, corresponding to diploid(2c), tetraploid(4c), and octaploid(8c). These proportions changed in the course of regeneration which were clearly demonstrated by DNA histograms using flow cytometry. The proportion of diploid, tetraploid, and octaploid nuclei in control liver were 49.3±1.6%, 45.0±7.4%, and 1.7±0.7%, respectively. A significant change occurred at 24 hours after hepatectomy, as FCM revealed 25.9±1.1% diploid, 54.5±1.2% tetraploid, and 9.0±0.9% octaploid. This shift to polyploid nuclei persisted until 72 hours, and then gradually returned to the pattern of control liver. The S-phase nuclei which incorporated BrdU increased rapidly at 24 hours to a peak of 11.3±0.9%, and gradually decreased to 5.8±0.8%, 5.3±0.8%, 2.4±0.6%, 2.9±1.1%, and 1.2±0.6%, at 48, 72, 96, 120, and 168 hours, respectively. This 2-color FCM analysis made a detailed analysis of the cell kinetics in regenerating hepatocytes possible, and may be applied in investigations of various aspects of liver regeneration.

KEY WORDS: Flow cytometry, liver regeneration, cell cycle, bromodeoxyuridine

### INTRODUCTION

Liver regeneration is an extraordinary phenomena the mechanism of which has been investigated from many points of view over a long period. In terms of hepatotrophic factors, the significance of portal blood flow was stressed,[1-3] and insulin and glucagon were shown to be portal humoral factors for liver regeneration[4-6]. Many non-portal factors were also investigated, including thyroid hormone,[7] glucocorticoids,[8] prostaglandins,[9] and epidermal growth factor[10].

In addition to hepatotrophic factors, there are many problems to be solved in liver regeneration from the point of view of molecular biology and genetics. For example, the mechanism of initiation and termination of liver regeneration, as well as the interaction between parenchymal and non-parenchymal liver cells. The latter are known to produce hepatocyte growth factor (HGF)[11] or transforming growth factor $\beta$[12] this indicates a possible paracrine mechanism of growth regulation, which is yet to, and be investigated. It is clinically important in surgery to appreciate the cell kinetics after partial hepatectomy and also the recently introduced transhepatic embolization prior to radical operation[13].

Generally, the cycle of proliferating cells is divided into four stages $G_1$, S, $G_2$, and M phase. S phase and M phase are characterized by DNA synthesis, and mitosis, respectively, $G_1$ and $G_2$ are phases between these two stages. There is another stage which is the non-proliferating state of cells - $G_0$ phase. In normal liver, hepatocytes are in $G_0$ phase. When some induction to regeneration (for example, partial hepatectomy) occurs, hepatocytes enter $G_1$ phase, which is an initial stage in cell cycle. They proceed through the cell cycle ($G_1$-S-$G_2$-M), and at the end of M phase, cell division occurs. Normally, $G_0/G_1$ phase is characterized by 2n (diploid), $G_2$/M phase by 4n (tetraploid), and S phase by somewhere between 2n and 4n. However, there are a large proportion of polyploid nuclei in hepatocytes, and they are tetraploid or occasionally octaploid (8n) at $G_0$ phase.

Recently, 2-color flow cytometric(FCM) analysis has been applied to the investigation of cell kinetics[14,15]. The intensity of red fluorescence of propidium iodide (PI), a DNA staining dye, is in proportion to the amount of DNA in cell nuclei when they are stained with the dye. The relative DNA content of each nuclei (i.e. cell nuclei ploidy) can then be measured by flow cytometry. Diploid, tetraploid, and octaploid cell nuclei show different peaks in DNA histograms, in parallel with their DNA content. In addition, bromodeoxyuridine(BrdU), a thymidine analogue, is uptaken into DNA during DNA synthesis (i.e. S phase). Because BrdU in cell nuclei is detectable by staining with fluorescein isothiocyanate(FITC) conjugated anti-BrdU monoclonal antibody, it is possible to discriminate S phase nuclei from others using FCM. By 2-color FCM analysis with propidium iodide and FITC conjugated anti-BrdU MoAb, the fluorescence of PI(red) and FITC(green) in each cell is measured independently. Each phase of the cell cycle - $G_0/G_1$, S, and $G_2$/M phase - is then clearly recognized in a cell population. This method is simple, free of radiotoxicity, and a large number of cells is analyzed in seconds.

There are mononuclear and binuclear cells amongst mammalian hepatocytes. There are also polyploid nuclei such as tetraploid or octaploid, in addition to diploid, and the proportions of these change in the course of liver regeneration[16]. However, this has not been fully investigated. Because of the complexity of hepatocyte DNA histogram patterns and cell cycles, FCM analysis is suitable for investigating the cell kinetic state of liver regeneration. In this series, we applied this method to investigate the cell kinetics of regenerating liver after partial hepatectomy in rats.

### METHODS

### *Animals*

Adult male Wistar rats weighing 250-300 grams were purchased from SLC Co.Ltd.(Shizuoka, Japan). They were fed a standard pellet rat diet *ad libitum* pre- and postoperatively and maintained on 12 hour light-dark cycles. A 70% partial hepatectomy was performed in the morning under light ether anesthesia, according to the technique of Higgins and Anderson[17]. Sham operation consisted of laparotomy and cutting of the falciform ligament of the liver.

### *BrdU Labeling and Liver Perfusion*

20mg/kg of 5-bromo-2′-deoxyuridine (BrdU: Sigma, St. Louis, USA) was injected through the penile vein as a pulse label for detection of S phase hepatocytes in regenerating liver, at given time intervals (6, 12, 24, 48, 72, 96, 120, and 168 hours) after operation. Rats were anesthetized one hour after the injection using intraperitoneal pentobarbital sodium (30mg/kg of body weight). Liver cells were isolated by the collagenase perfusion method, previously described by Seglen[18]. Briefly, the portal vein was cannulated with a polyethylene catheter, and the superior vena cava was cut for drainage. Immediately after cannulation, 100ml of 5mM EGTA-Hepes buffer solution was perfused for about 5 minutes, followed by 200-250ml of 0.05% collagenase (Wako Pure Chemical Industries, Osaka, Japan)-Hepes buffer solution to disperse the tissue. The liver was then removed, dispersed by surgical blades in cold $Ca^{++}$ and $Mg^{++}$ free phosphate buffered saline (PBS(-)), filtered through gauze, and centrifuged several times at 50g for 5 minutes in cold PBS(-) to isolate the hepatocytes. The samples were then fixed with 70% ethanol, and stored at -20°C until the assay.

*Sample Preparation for FCM Analysis*

Samples were prepared for 2-color FCM analysis using anti-BrdU monoclonal antibody according to a modification of the method described previously[14,15]. Briefly, samples were suspended in 4N HCl for 20 minutes for partial strandization of DNA. This is necessary in order for the monoclonal antibody used in this series to gain access to incorporated BrdU in the DNA. The solution is then neutralized with 0.1M sodium tetraborate. After washing with cold PBS(-), hepatocytes were suspended in 0.1% pepsin (Sigma, adjusted to pH 1.5 with 6N HCl) for 5 minutes at room temperature to isolate nuclei, as shown in Figure 1. They were then washed with cold PBS(-) twice by centrifuging at 500g for 10 minutes, and the number of nuclei were adjusted to $1 \times 10^6$. To each pellet of sample, 20$\mu$l of fluorescein isothiocyanate (FITC) conjugated anti-BrdU monoclonal antibody (Becton-Dickinson, Mountain View, USA) was added and incubated for 20 minutes at room temperature. Nuclei were then washed twice in cold PBS(-), and finally the pellet was suspended in 2ml of propidium iodide (PI) solution containing 5$\mu$g of PI per ml. Samples were stored on ice in the dark for at least 1 hour. They were filtered using a 20$\mu$m nylon mesh immediately prior to FCM analysis.

*Flow Cytometry Analysis*

The fluorescence emission of the stained nuclei was measured on a FACS 440 flow cytometer/cell sorter (Becton-Dickinson). The green fluorescence was monitored using the 530nm band pass filter. Red fluorescence was observed after a 640nm long pass filter. The excitation source consisted of an argon-ion laser operated at the 488nm line with 300mW power output. A minimum of $5 \times 10^4$ nuclei were evaluated for each sample in dual parameter analysis of DNA content and BrdU uptake. The proportion of each ploidy of isolated nuclei and those that were labeled by FITC conjugated monoclonal antibody were evaluated.

*Total DNA Content of Regenerating Liver*

The DNA content of regenerating livers, at designated time intervals after

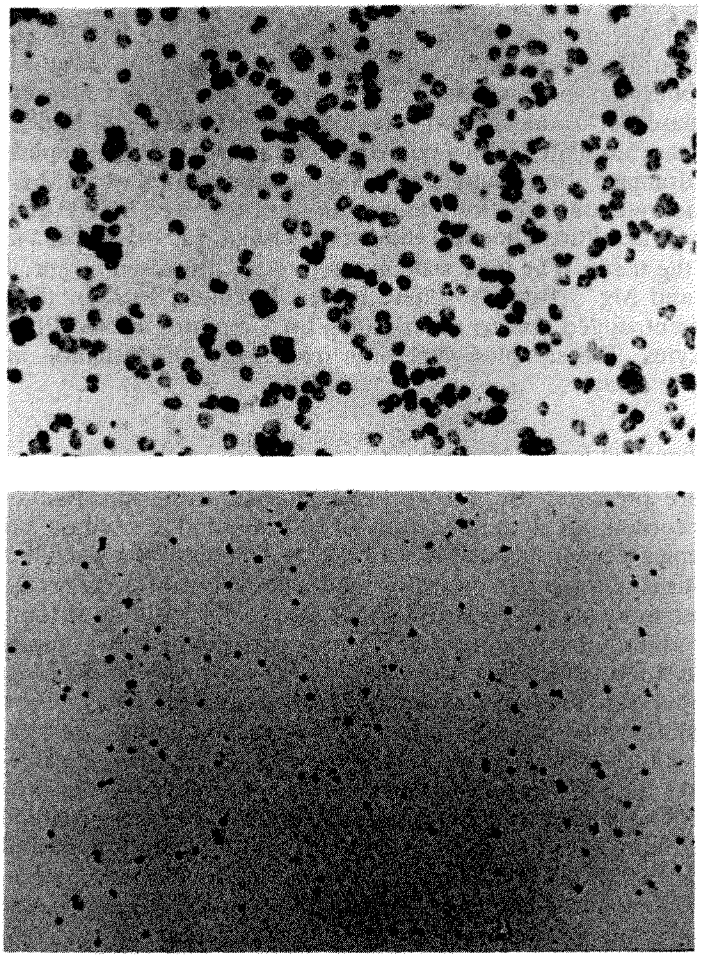

(a)

(b)

**Figure 1** Photomicrographs of isolated hepatocytes and hepatocyte nuclei. (a) Hepatocytes isolated with the collagenase perfusion method and fixed with 70% ethanol. ×200. (b) Hepatocyte nuclei extracted by 0.1% pepsin treatment. Hematoxylin stain. ×200.

hepatectomy, were measured by the diphenylamine reaction, according to the method of Burton[19].

*Statistical Analysis*

Student's $t$ test for nonpaired samples was used for statistical analysis, and statistical significance was reached when the $p$ value was less than 0.05. Results were expressed as mean ± S.E.

### RESULTS

Figure 2 shows representative DNA histograms (A) and contour graphs (B) of hepatocyte nuclei at designated time intervals after partial hepatectomy. The DNA histogram showed two peaks of diploid and tetraploid in controls. There was no measurable octaploid nuclei peak. The 4c/2c ratio (the ratio of tetraploid nuclei to diploid) was 0.91. Contour graphs showed that only a few nuclei incorporated BrdU. At 6 and 12 hours, no significant changes occurred either in the histograms

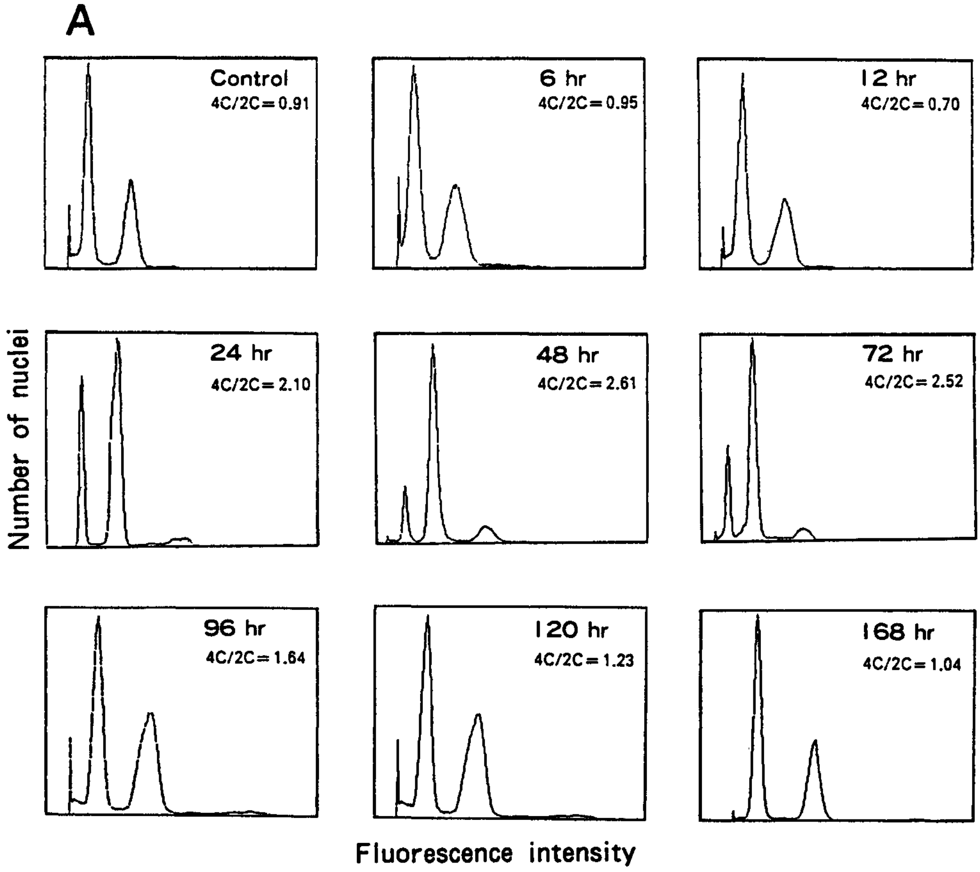

**Figure 2** Representative DNA histograms (A) and Contour graphs (B) at time intervals after 70% hepatectomy. Fluorescence staining was performed as described in methods. In (A), X and Y axis represent fluorescence intensity of PI (linear scale) and cell number, respectively. As PI intensity is in proportion to the DNA content in each nuclei, each peak shows an accumulation of diploid(2c), tetraploid(4c), and in some cases, octaploid(8c) nuclei. In (B), X and Y axis represent PI intensity (linear scale) and FITC intensity (log scale), respectively. FITC intensity of anti-BrdU MoAb shows the amount of BrdU uptaken into DNA, which is in proportion to the rate of DNA synthesis in each nuclei. DNA synthesizing (S phase) cell nuclei, which is BrdU positive, appear in upper part of the graph. The contour shows the number of nuclei. PI:propidium iodide, FITC:fluorescein isothiocyanate MoAb;monoclonal antibody.

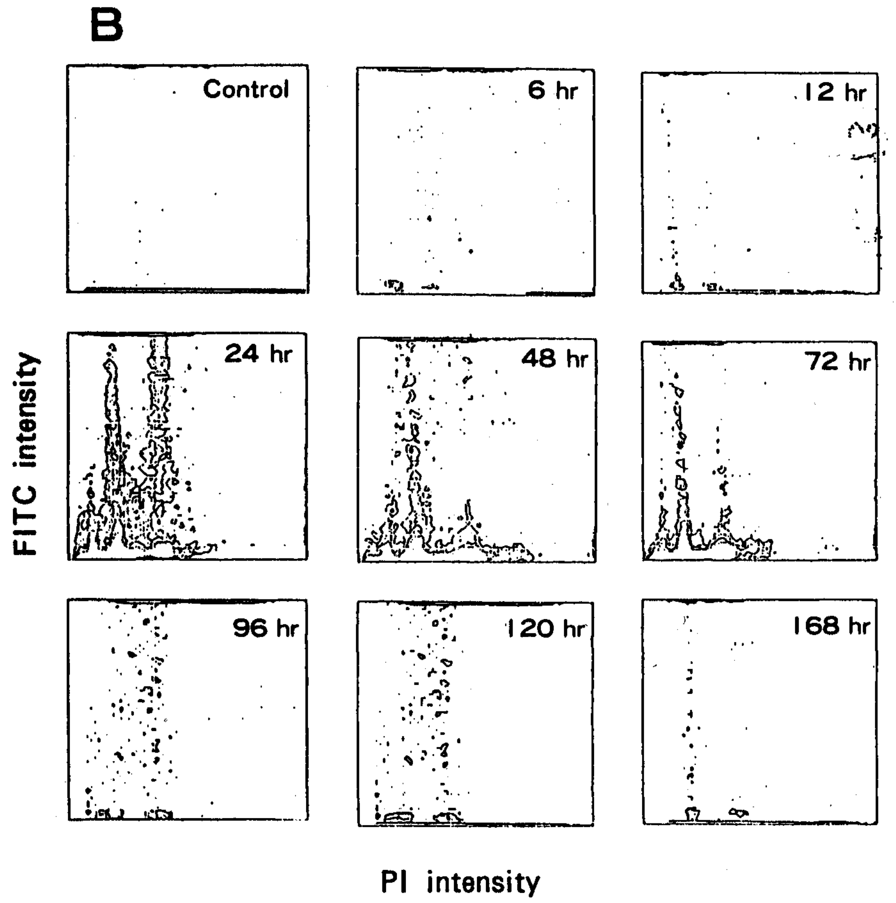

**Figure 2** (*continued*)

or contour graphs. At 24 hours, the DNA histogram patterns changed so that the 4c/2c ratio increased, and the number of 8c (octaploid) nuclei also increased to create the apparent third peak. This polyploidization proceeded during an initial phase of regeneration, with a maximum 4c/2c ratio (2.61) at 48 hours. The pattern then gradually recovered to that of the control, and the 4c/2c ratio decreased to 1.04 at 168 hours, which was nearly the level of the control. Contour graphs at 24 hours demonstrated the appearance of S phase nuclei, which incorporated BrdU into DNA. BrdU incorporated nuclei were seen between diploid and tetraploid, and also tetraploid and octaploid in contour graphs. The number of BrdU incorporated nuclei gradually decreased after 24 hours, and a few nuclei were BrdU positive at 168 hours (1.2 ± 0.6%).

Figure 3 shows changes in the rate of diploid, tetraploid, and octaploid nuclei at time intervals after 70% hepatectomy. In controls, the rate of diploid, tetraploid, and octaploid were 49.3 ± 1.6%, 45.0 ± 7.4%, and 1.7 ± 0.7%, respectively. At 12

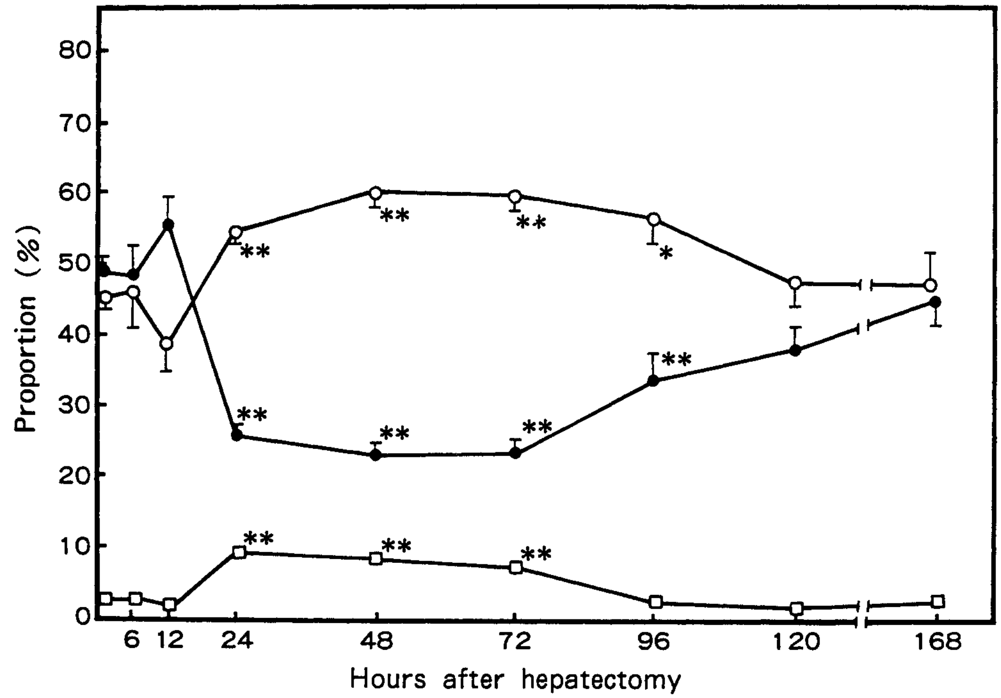

**Figure 3** Changes in the proportion of diploid (closed circles), tetraploid (open circles), and octaploid (open squares) nuclei at time intervals after 70% hepatectomy. The proportion of each ploidy was calculated from DNA histograms using Consort 30 (Becton-Dickinson) (mean ± S.E.). N = 5, 8, 9, 10, 10, 10, 5, 5, and 5 at 0, 6, 12, 24, 48, 72, 96, 120, and 168 hours respectively. * and **: Significantly different from the value of each ploidy at 0 hours ($P<0.05$ and $P<0.01$, respectively).

hours, diploid nuclei increased to 55.8 ± 3.6%, and tetraploid decreased to 38.7 ± 4.1%, which were not significantly different from controls. At 24 hours, diploid nuclei decreased to 25.9 ± 1.1%, while tetraploid and octaploid nuclei increased to 54.5 ± 1.2% and 9.0 ± 0.9%, respectively. This polyploidization persisted until 72 hours, when the proportion of each ploidy gradually recovered. At 7 days after hepatectomy, the proportions were 44.8 ± 7.0% diploid, 46.8 ± 5.8% tetraploid, and 2.2 ± 0.6% octaploid nuclei, which were nearly the levels of control liver.

The proportion of S phase nuclei incorporating BrdU at time intervals after 70% hepatectomy is shown in Figure 4. Labeling index was highest at 24 hours, 11.3 ± 0.9%, and then gradually decreased to 5.8 ± 0.8%, 5.3 ± 0.8%, 2.4 ± 0.6%, 2.9 ± 1.1%, and 1.2 ± 0.6% at 48, 72, 96, 120, and 168 hours, respectively. The number of samples were 5, 8, 9, 10, 10, 10, 5, 5, and 5 at 0, 6, 12, 24, 48, 72, 96, 120, and 168 hours, respectively in Figures 3 and 4.

Figure 5 shows the recovery rate of total DNA content of regenerating liver after 70% hepatectomy. The recovery rate at each time interval was 35.4 ± 4.9%, 48.5 ± 2.7%, 66.8 ± 10.7%, and 89.1 ± 7.2% at 24, 48, 72 and 168 hours, respectively. The number of samples were 5 at each time interval.

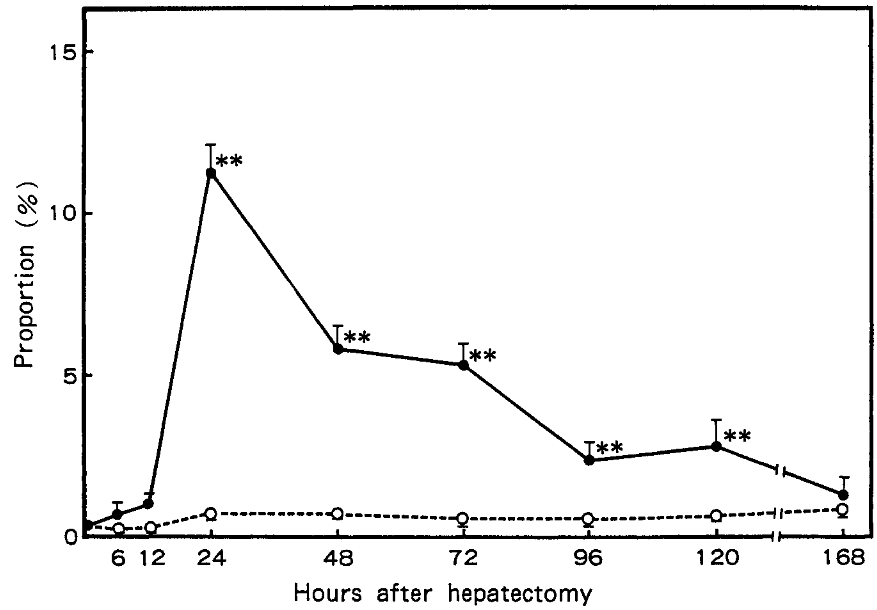

**Figure 4** Changes in the proportion of S-phase nuclei incorporated BrdU into DNA at time intervals after 70% hepatectomy. The proportion was calculated from single parameter histogram of FITC intensity using Consort 30 (Becton-Dickinson) (mean ± S.E.). The number of samples at each time interval are described in Figure 3. N = 3 at each time interval in sham group. * and **: Significantly different from control ($P<0.05$ and $P<0.01$, respectively).

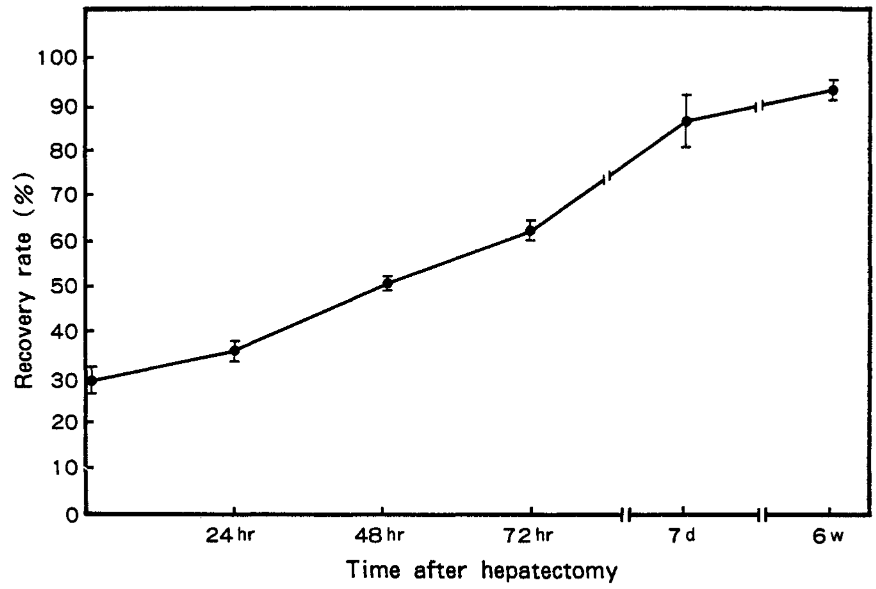

**Figure 5** Recovery rate of the total DNA content of regenerating liver at postoperative time intervals (mean ± S.E.). The 100% value is the estimated total DNA content of preoperative liver, calculating from the DNA content of the resected liver as being 70%. N = 5 at each time interval.

### DISCUSSION

Seventy percent partial hepatectomy in rats has long been used as a model of liver regeneration. There are several methods for assessment of regeneration, including $^3$H-thymidine uptake, autoradiography, and mitotic index in histology, as well as the labeling index in immunohistochemistry using proliferative markers such as bromodeoxyuridine, calculating the percentage of stained (BrdU-positive) cells among whole cells by microscopic examination.

Two-color flow cytometric (FCM) analysis using monoclonal antibody to bromodeoxyuridine[14] and DNA staining dye such as propidium iodide, is a useful method for investigating cell kinetics[15]. There are several reasons for this: 1) the method is free of radiotoxicity, 2) each phase of the cell cycle (e.g. $G_0/G_1$, S, and $G_2/M$ phase) can be clearly discriminated, 3) several fold of $10^4$ cells can be measured in seconds, and 4) the procedure is relatively simple. As described previously, the distribution of ploidy seen in parenchymal cells of mammalian liver is complex,[16] that is, mononuclear and binuclear cells, and we observed diploid, tetraploid and octaploid nuclei in DNA histograms (Figure 2A). Therefore, there are two cell cycles seen during regeneration (Figure 2B). FCM analysis is a useful method to investigate this complicated ploidy distribution and cell kinetics of hepatocytes in the course of proliferation. There are a few reports of flow cytometric analysis of hepatocytes in rats and mice,[20-22] investigating the effect of carcinogen treatment on ploidy distribution, its difference among strains, and its change with aging. Digernes *et al.* assessed DNA synthesis in rat hepatocytes after 70% hepatectomy by flow cytometric determination of nuclear DNA content and by incorporation of $^3$H-thymidine, finding a fairly good correlation between the two different methods[23]. However, two-color FCM analysis using BrdU monoclonal antibody, a more accurate and direct method of determining the S phase fraction among proliferating cells, has not been used to investigate liver regeneration.

At 12 hours after hepatectomy, the proportion of each ploidy changed slightly, but not significantly (Figures 2a, 3). The significant change in ploidy distribution occurred at 24 hours after hepatectomy, together with the initiation of DNA synthesis (Figure 4), in such a way that tetraploid became dominant instead of diploid (4c/2c ratio changed from 0.70 to 2.10), and octaploid appeared to make an apparent peak in the DNA histogram (Figures 2a, 3). These proportional changes persisted until 72 hours, which indicates that the rate of DNA synthesis and nuclear division maintain equilibrium during the 24-48 hours after hepatectomy. After 72 hours, this equilibrium changed so that nuclear division dominated, and the ploidy patterns gradually returned to that of control liver. These findings indicate that the initiation of DNA synthesis varies among hepatocytes, probably due to the topographical difference of each cell in its hepatic acinus, which is the structural and functional unit of liver parenchyma and is composed of hepatocytes differing in their functions,[24,25] although the sharp peak is seen at 24 hours. This is ascertained by the recovery rate of total DNA content of regenerating liver (Figure 5), which gradually increased after 24 hours, and nearly reached baseline at 168 hours (89.1 ± 7.2%).

There is a rapid rise in the rate of DNA synthesizing nuclei to a peak at 24 hours after 70% hepatectomy, followed by a relatively sharp decline. As mentioned previously, BrdU incorporated nuclei were seen between diploid and tetraploid, and tetraploid and octaploid, as clearly demonstrated in contour graphs at 24 hours

(Figure 2b). This indicates DNA synthesis is initiated in both diploid and tetraploid nuclei at the same time during liver regeneration. A few S phase nuclei were seen at 7 days after hepatectomy. These patterns of DNA synthesis are similar to the results obtained with $^{3}$H-thymidine uptake, described previously,[25,26] although the labelling index (L.I.) is higher in autoradiography. The difference in L.I. may be partly because the rats were relatively mature, being more than 10 weeks old in this series. Another possible reason is that we stained BrdU in hepatocyte nuclei by the direct method using fluorescein conjugated anti-BrdU MoAb. The labelling index may be higher by the indirect method, using both primary and secondary antibodies to increase sensitivity.

The conventional method for quantitative evaluation of liver regeneration has been a measurement of $^{3}$H-thymidine uptake, however, this only shows the rate of DNA synthesis as a liver mass, and it is impossible to observe the whole cell cycle by this method. Two color flow cytometry, investigating both the total DNA content and BrdU uptake of each nuclei, has made it possible to describe whole cell kinetics of hepatocytes during regeneration.

There are a few investigations concerning the significance of polyploidy in parenchymal cells of the liver[27,28]. For example, it is speculated that there is a mutual balance between tissue specific functions and proliferative response, and polyploidy appears when the former dominates over the latter[27]. Another hypothesis of the biological significance of liver cell polyploidy is that polyploidization ensures protection against the deleterious consequences of aberrant genome formation resulting from aberrant mitosis[28]. However, a functional difference has not been clearly demonstrated between diploid nuclei (cells) and other polyploid nuclei (cells). As for DNA synthesis, diploid nuclei may be easier to get into the cell cycle (i.e. begin DNA synthesis) than are tetraploid nuclei, because at 24 hours, the decrease in diploid nuclei was much greater than the increase in octaploid nuclei (Figure 3). Cell selection by a cell sorter equipped with flow cytometry would make it possible to investigate the different functions of cells of each ploidy.

In conclusion, 2-color FCM analysis, a new method for detecting cell kinetics, is also applicable to investigating liver regeneration, and provides more detailed information as to cell cycle than have previous methods.

*Acknowledgements*

We wish to thank Katsuyuki Ohmori and Tomoya Yoneda for their skillful technical assistance in flow cytometric analysis, and Katsujiro Manabe, M.D., Minoru Nakatake, M.D. for their support. This work was partly supported by Grant-in-Aid for Developmental Scientific Research (C) from the Ministry of Education, Science and Culture (Grant No. £63570629).

*References*

1. Fisher, B., Szuch, P. and Fisher, E.R. (1971) Evaluation of a humoral factor in liver regeneration utilizing liver transplants. *Cancer Research*, **31**, 322–330

2. Rous, P. and Larimore, L.D. (1920) Relation of the portal blood to liver maintenance. *Journal of Experimental Medicine*, **31**, 609–632
3. Starzl, T.E., Francavilla, A., Halgrimson, G.G., Francavilla, F.R., Porter, K.A., Brown, T.H. and Putnum, C.W. (1973) The origin, hormonal nature, and action of hepatotrophic substances in portal venous blood. *Surgery, Gynecology and Obstetrics*, **137**, 179–199
4. Leffert, H.L. (1974) Growth control of differenciated fetal rat hepatocytes in primary monolayer culture. Hormonal control of DNA synthesis and its possible significance to the problem of liver regeneration. *Journal of Cell Biology*, **62**, 792–801
5. Whittemore, A.D., Kasuya, M., Voorhees, A.B. and Price, J.B. (1975) Hepatic regeneration in the absence of portal viscera. *Surgery*, **77**, 419–426
6. Bucher, N.L.R. and Swaffield, N.N. (1975) Regulation of hepatic regeneration in rats by synergistic action of insulin and glucagon. *Proceedings of the National Academy of Sciences of the U.S.A.*, **72**, 1157–1160
7. Leffert, H.L. and Alexander, N.M. (1976) Thyroid hormone metabolism during liver regeneration in rats. *Endocrinology*, **98**, 1241–1247
8. Leffert, H.L., Moran, T., Boorstein, R. and Koch, K.S. (1977) Procarcinogen activation and hormonal control of cell proliferation in differenciated primary adult rat liver cell cultures. *Nature*, **267**, 58–61
9. Richmann, R.A., Clans, T.H., Pilkis, S.J.P. and Friedman, D.L. (1976) Hormonal stimulation of DNA synthesis in primary culture of adult rat hepatocytes. *Proceedings of the National Academy of Sciences of the U.S.A.*, **73**, 3589–3593
10. Kanzaki, Y., Mahmud, I., Asanagi, M., Fukui, N. and Miura, Y. (1979) Thromboxane as a trigger for liver regeneration. *Cell and Molecular Biology*, **25**, 147–152
11. Kinoshita, T., Tashiro, K. and Nakamura, T. (1989) Marked increase of HGF mRNA in non-parenchymal liver cells of rats treated with hepatotoxins. *Biochemical and biophysical research communications*, **165**, 1229–1234
12. Braun, L., Mead, J.E., Panzica, M., Mikumo, R., Bell, G.I. and Fausto, N. (1988) Transforming growth factor $\beta$ mRNA increases during liver regeneration: A possible paracrine mechanism of growth regulation. *Proceedings of the National Academy of Sciences of the U.S.A.*, **85**, 1539–1543
13. Makuuchi, M., Thai, B.L., Takayasu, K., Takayama, T., Kosuge, T., Gunven, P., Yamazaki, S., Hasegawa, H. and Ozaki, H. (1990) Preoperative portal embolization to increase safety of major hepatectomy for hilar bile duct carcinoma: A preliminary report. *Surgery*, **107**, 521–527
14. Gratzner, H.G. (1982) Monoclonal antibody to 5-Bromo- and 5-Iododeoxyuridine: A New Reagent for Detection of DNA Replication. *Science*, **218**, 474–475
15. Dolbeare, F., Gratzner, H.G., Pallavicini, M.G. and Gray, J.W. (1983) Flow cytometric measurement of total DNA content and incorporated bromodeoxyuridine. *Proceedings of the National Academy of Sciences of the U.S.A.*, **80**, 5573–5580
16. Bucher, N.L.R. (1963) Regeneration of Mammalian Liver. *International Review of Cytology*, **15**, 245–300
17. Higgins, G.M. and Anderson, R.M. (1931) Experimental pathology of the liver. *Archives of Pathology*, **12**, 186–202
18. Seglen, P.O. (1976) Preparation of isolated liver cells. *Methods in Cell Biology*, **13**, 29–83
19. Burton, K. (1956) A study of the conditions and mechanism of the diphenylamine reaction for the colorimetric estimation of deoxyribonucleic acid. *Biochemistry*, **62**, 315–323
20. Severin, E., Willers, R. and Bettecken, T. (1984) Flow cytometric analysis of mouse hepatocyte ploidy. II. The development of polyploidy pattern in four mice strains with different life spans. *Cell and Tissue Research*, **238**, 649–652
21. Sater, G., Schwarze, P.E. and Seglen, P.O. (1988) Shift From Polyploidizing to Nonpolyploidizing Growth in Carcinogen-Treated Rat Liver. *Journal of the National Cancer Institute*, **80**, 950–957
22. Danielsen, H.E., Steen, H.B., Lindmo, T. and Reith, A. (1988) Ploidy distribution in experimental liver carcinogenesis in mice. *Carcinogenesis*, **9**, 59–63
23. Digernes, V., Bronstad, G., Sand, T.E. and Christoffersen, T. (1982) The proliferative response of rat liver parenchymal cells after partial hepatectomy. *Cell and Tissue Kinetics*, **15**, 521–528
24. Rappaport, A.M. (1973) The microcirculatory hepatic unit. *Microvascular Research*, **6**, 212–228
25. Hartmut, M.R. (1976) Kinetics of hepatocellular proliferation after partial resection of the liver. Edited by H. Popper and F. Schaffner: *Progress in Liver Diseases*, pp. 83–99 Vol. V. New York: Grune & Stratton
26. Grisham, J.W. (1962) A morphologic study of deoxyribonucleic acid synthesis and cell proliferation in regenerating rat liver; autoradiography with thymidine-$^3$H. *Cancer Research*, **22**, 842–849

27. Brodsky, W.Y. and Uryvaeva, I.V. (1977) Cell polyploidy: Its relation to tissue growth and function. *International Review of Cytology*, **50**, 275–332
28. Uryvaeva, I.V. (1981) Biological significance of liver cell polyploidy: an Hypothesis. *Journal of Theoretical Biology*, **89**, 557–571

*(Accepted by S. Bengmark on 26 June 1991)*

### INVITED COMMENTARY

Proliferation and differentiation are the essential manifestations of living organisms. Mammalian cells generally express these exquisitely controlled properties reciprocally. Mature tissues in higher animals contain cell populations whose specialized functions are expressed optimally during proliferative quiescence. With respect to hepatocyte growth quiescence does not imply a zero proliferation rate. There is a low frequency of differentiated hepatocyte entry into the cell cycle that is balanced by low rates of hepatocyte aging and death. The cell cycle concept is useful to quantitate kinetic changes as cells multiply. Upon stimulation proliferation increases 600-fold. In the regenerating rat liver most hepatocytes proliferate at least once within 24–36 hours. The liver proliferative response is elegantly controlled and the growth will eventually cease. Knowledge of the factors that control this proliferative response both at initiation and at termination will be of utmost importance for the study of cell cycle control in general and in carcinogenesis. The quantitative evaluation of liver regeneration has so far mainly been limited to measurements of $^3$H-thymidine uptake which only measures the S phase. With the 2-color flow cytometry presented in this elegant study it will be possible to describe whole cell kinetics of hepatocytes during regeneration. It will give us an excellent opportunity to study influences from different sources during different parts of the cell cycle during proliferation. It will also give us the possibility of studying the effect of cellular regeneration after different noxious insults to the liver. We are now looking forward to results from the use of this technique to clarify the mechanisms of cellular response essential to HPB surgeons.

Bengt Jeppsson
Department of Surgery
Lund University
Lund, Sweden

### INVITED COMMENTARY

Analysis of cell cycle kinetics in the living organism is a difficult and challenging task. Flow cytometric techniques using DNA dyes like propidium iodide and bromodeoxyuridine incorporation into DNA offer useful insights. The cell cycle kinetics of most tissues such as normal gut mucosa conform to the classic cell cycle concept of DNA turnover. That is a 2c DNA content in the $G_0/G_1$ phase, 4c DNA content in the $G_2M$ phase and intermediate DNA values in the S phase.

By contrast hepatocytes appear to exhibit true polyploidy with significant tetraploid (4c) and octaploid (8c) cell populations. Careful interpretation of DNA histograms is required to confirm true polyploidy, as a spurious result can occur from simple clumping of cell nuclei — in which case 6c and 10c peaks may also be identified in the histogram.

This account of a dynamic shift in the ploidy distribution of regenerating hepatocytes is particularly fascinating. It appears to show that polyploidy is a definite part of the physiological proliferating cycle of hepatocytes and not an isolated curiosity. Some biological advantage must exist for the hepatocyte, to preferentially develop within the cell, a nucleus that may contain twice (tetraploid) or four times (octaploid) the normal DNA content. What this advantage may be and why this phenomena is not present in epithelial tissues is not clear. However, further studies using two colour flow cytometric analysis in modals of abnormal hepatocyte proliferastion (e.g. cirrhosis), may elucidate the significance of polyploidisation in the regenerating liver.

Nigel A. Scott
Department of Surgery
UWCM
Cardiff, UK

